# Senescent Atrophic Epidermis Retains Lrig1+ Stem Cells and Loses Wnt Signaling, a Phenotype Shared with CD44KO Mice

**DOI:** 10.1371/journal.pone.0169452

**Published:** 2017-01-18

**Authors:** Laurent Barnes, Jean-Hilaire Saurat, Gürkan Kaya

**Affiliations:** 1 Department of Dermatology, University Hospital of Geneva, University of Geneva, Geneva, Switzerland; 2 Swiss Center for Applied Human Toxicology, University of Geneva, Geneva, Switzerland; NYU Langone Medical Center, UNITED STATES

## Abstract

Lrig1 is known to repress the epidermal growth through its inhibitory activity on EGFR, while CD44 promotes it. We analyzed the expression of these molecules in senescent atrophic human epidermis and in the epidermis of CD44KO mice. In normal human epidermis, Lrig1+ cells form clusters located in the basal layer in which CD44 expression is downregulated and Lef1 expression reflects an active Wnt signaling. In senescent atrophic human epidermis, we found retention of Lrig1^high^+ cells all along the basal layer, forming no clusters, with decrease of CD44 and lef1 expression. In vitro silencing of CD44 indicated that CD44 may be required for Wnt signaling. However, if looking at the ear epidermis of CD44KO mice, we only found a limited interfollicular epidermal atrophy and unchanged Lrig1^high^+ cells in the hair follicle. Cell lineage tracing further revealed that interfollicular epidermis did lost its self-renewing capacity but that its homeostasis relied on Lrig1-derived keratinocytes migrating from the hair follicle. Therefore, we conclude that CD44 downregulation is part of the phenotype of senescent atrophic human epidermis, and contributes to reduce Wnt signaling and to alter Lrig1^high^+ stem cell distribution.

## Introduction

In mice, the epidermis is divided into different compartments, each one self-renewing independently using its own niche of stem cells [1,2,3]. The interfollicular epidermis (IFE) appears to be an independent compartment which shows self-renewal as basal keratinocytes divide according to an asymmetric model of division [4,5]. The isthmus and junctional zone, where Lrig1 is expressed, contains the stem cells that renew in homeostasis the infundibulum, junctional zone and the sebaceous gland, while keratinocytes expressing CD34 and Lgr5 in the bulge region of the hair follicle are considered to be the hair follicle stem cells [2]. However, cells from the hair follicle are able to renew all the epidermal compartments upon injury [2,6].

In human skin, such a compartmentalization can hardly be demonstrated. Lrig1 has been reported to be expressed in the human IFE basal layer preferentially in clusters of keratinocytes located on the top of rete ridges [7]. This is a substantial difference with the murine model, where Lrig1 is expressed in the isthmus only and is not detected in the IFE. This might be related to the fact that in human epidermis, the IFE is much larger than in mouse epidermis. However, recently an Lrig1+ cell cluster has also been identified in the isthmus of the human hair follicle [8].

The biological function of Lrig1 resides in its inhibitory activity for the epidermal growth factor receptor (EGFR) [9,10]. This receptor promotes epidermal growth and forms with Lrig1 an auto-regulatory loop that is assumed to regulate the epidermal homeostasis [7]. Lrig1 knock-down induces epidermal hyperplasia, confirming its growth inhibitory activity. This is consistent with a similar biological activity also described in the intestinal epithelium [11,12]. Cell surface receptor of hyaluronate (HA), CD44, was reported to be crucial for epidermal homeostasis [13]. The importance of CD44 in the epidermis may be related to its role in the putative molecular platform, hyalurosome, where it modulates the activity of EGFR and F-actin cytoskeleton [13,14,15]. CD44 downregulation drives the atrophy in mouse epidermis induced by corticosteroids and is associated with human epidermal atrophy [13,14,16]. This kind of severe form of epidermal atrophy was described in humans as part of the phenotype of a general syndrome of skin insufficiency associated with aging and topical and systemic corticosteroid treatments, also called dermatoporosis [8,16,17]. The CD44 knock-out (CD44KO) mice however do not show a significant skin phenotype consistent with the major function of CD44 in epidermal homeostasis [18], apart from some alterations in keratinocyte differentiation and epidermal permeability [19,20]. Transgenic mice where the expression of CD44 was selectively suppressed in the epidermis showed an epidermal atrophy with accumulation of HA in the superficial dermis [21].

To better understand the role of Lrig1+ stem cells in epidermal atrophy, we tracked the Lrig1-derived keratinocytes in the ear epidermis of CD44KO mice and investigated the expression of Lrig1, EGFR, CD44v3, a splice variant of CD44 expressed in keratinocytes, and the molecules of Wnt pathway in normal and senescent atrophic human epidermis (SAHE).

## Materials & Methods

### Animals

CD44KO mice (B6.129(Cg)-cd44^tm1Hbg^/J) (strain 1) were purchased from Jackson Laboratory (Bar Harbor, M, USA), as well as B6.129S6(Cg)-Lrig1^tm1.1(cre/ERT2)rjc^/J (strain 2) and the B6;129S6-Gt(ROSA)26Sor^tm9(CAG-tdTomato)Hze^/J (strain 3) with respective Jaxmice reference: 005085, 018418 and 007905. Strain 1 and 2 were crossed to obtain CD44KO animals hemizygote for the Lrig1:.CreER transgene (strain 4, Lrig1CreER/CD44KO). At the same time strain 1 and 3 were crossed to obtain homozygote animals for tomato and CD44KO (strain 5) and a new generation of animals only homozygote for tomato (strain 3’). To perform Lrig1 cell lineage tracing in a WT background, the strain 2 and 3’ were crossed to produce animals bearing the Lrig1CreER transgene and the Lox-stop-Lox tomato transgene. To perform Lrig1 cell lineage tracing in a CD44KO background, strain 4 was crossed with the strain 5 to obtain CD44KO animals bearing the Lrig1CreER transgene and the Lox-stop-Lox tomato transgene. Mice were genotyped with the mouse genotyping kit from KAPPABIOSYSTEMS (MA, USA) using the primers recommended by Jackson Laboratory. Tamoxifen was purchased from Sigma (T5648, Saint Louis, MI). WT and CD44KO transgenic mice expressing an inducible form of the Cre recombinase under the control of the Lrig1 promoter and a Cre reporter transgene coding for the tomato fluorescent protein were injected twice with 2mg of Tamoxifen in the first 24 hours in order to induce the recombinase activity and to remove the codon stop that prevents the constitutive expression of the tomato protein in Lrig1 expressing keratinocytes.

### Cell lineage tracing

Animals were killed at each timepoint after the Tamoxifen injections. Ears were fixed overnight in formalin and then included in Tissue-Tek OCT compound, Sakura Finetek Europe Alphen aan den Rijn, The Netherlands, and stored frozen. Skin sections were further prepared with a cryostat (-20°C), dried and mounted on histological slides with the DAPI fluoromount –G^(c)^ (SouthernBiotech Birmingham, AL, USA).

### Antibodies

Anti-Lrig1 (mouse) (R&D, AF3688, Minneapolis, MN, USA), anti-CD44 (mouse) (LifeSpan Biosciences, LS-C44149, Seattle, WA, USA), anti-EGFR (D38B1) XPTM polyclonal rabbit antibody (Cell Signaling, #4267, Danvers, MA, USA), F-actin staining with Phalloidin-Tetramethylrhodamine B isothiocyanate (SC 301530, Santa-Cruz Biotechnology^®^, Santa-Cruz, CA, USA), hyaluronate staining (B-HABP, 400763 Seikagaku Biobusiness Corporation, Tokyo, Japan), anti-keratin 5 (Rabbit polyclonal antibody keratin 5, AF138, Covance, NJ), anti-keratin 1 (Rabbit polyclonal antibody keratin 1, AF109; COVANCE, NJ). The Lrig1 antibody targeting the human Lrig1 was kindly provided by Dr. Satoshi Itami (Osaka, Japan) [12]. Anti-Lrig1 (western-blotting) (Cell Signaling, #17752, Danvers, MA, USA). Anti-CD44v3 (human CD44varv3 monoclonal antibody, BMS144, Bender MedSystems, Vienna, Austria). Anti-Lef1 (C12A5, rabbit monoclonal antibody, Cell Signaling, Danvers, MA, USA). Anti-β-catenin (D10A8 XP, rabbit monoclonal antibody, Cell Signaling, Danvers, MA, USA).

### Human skin samples

A group of 11 healthy skin biopsies and a group of 10 senescent atrophic skin samples, previously collected for medical diagnostic, were obtained from the histopathology collection of Dermatopathology Unit of the University Hospital of Geneva with the authorization CER12-091 of the Human Ethical Commission, University Hospital of Geneva. Some skin samples were re-excisions of the previously excised skin tumor wounds with no residual tumor. Skin samples were processed anonymously by the investigators. A trained dermatopathologist (GK) selected the atrophic skin samples in the group of aged patients.

### Immunostaining

Immunostaining studies were realized on the cryo-sections of mouse epidermis that were fixed in formalin before incubating with the respective antibodies. To visualize the tomato red fluorescent protein, skin sections from animals previously injected with Tamoxifen were fixed overnight in formalin. These sections were further embedded in OCT^®^ (Sakura, Japan) cryo-preservative medium, before making cryosections. Cryosections were further mounted in a Dapifluoromount-G^®^ (Southerntech, USA). Human histological slides were prepared from formalin-fixed paraffin-embedded sections. Slides were treated with citric acid (10mM, PH6) for antigen retrieval.

### Microscope

Images were obtained using a Leica SP5 confocal microscope.

### siRNA

siRNA interference experiments were performed by transfecting the cells with Lipofectamine^®^RNAiMAX and Opti-MEM^®^ from InvitrogenTM, Life Technologies according to the manufacturer’s instructions. Silencer Select pre-designed siRNAs for Lrig1 (s24973), CD44 (s2683) and a negative control (4390843) were purchased from Ambion^®^, Life Technologies. Keratinocytes were transfected and medium changed 24 hours after the transfection. At 48h, 50ng/mL of human recombinant Wnt-4 peptide (R&D, 6076-WN, Minneapolis, MN, USA) were added to the culture medium and cells were collected 24 hours later.

### Quantitative PCR

See S1 Text.

### Signal quantification

Image quantifications were performed with ImageJ software (NIH, USA).

### Statistics

ANOVA analyses were performed with GraphPad prism6, CA, USA.

### Ethics

This study was specifically approved by the Health Offices of Geneva and Ethical Committee on animal experimentation, and carried out in strict accordance with the recommendations of the Swiss animal experimentation recommendations in the animal facility of the University of Geneva (Permit number: GE/86/14). The protocol of animal experiments reported in this study was also revised and approved by Institutional Animal Care and Use Committee (IACUC) of Geneva University and carried out according their recommendations.

## Results

### Loss of CD44 gradient and retention of Lrig1+ stem cells in SAHE

**Fig 1:**
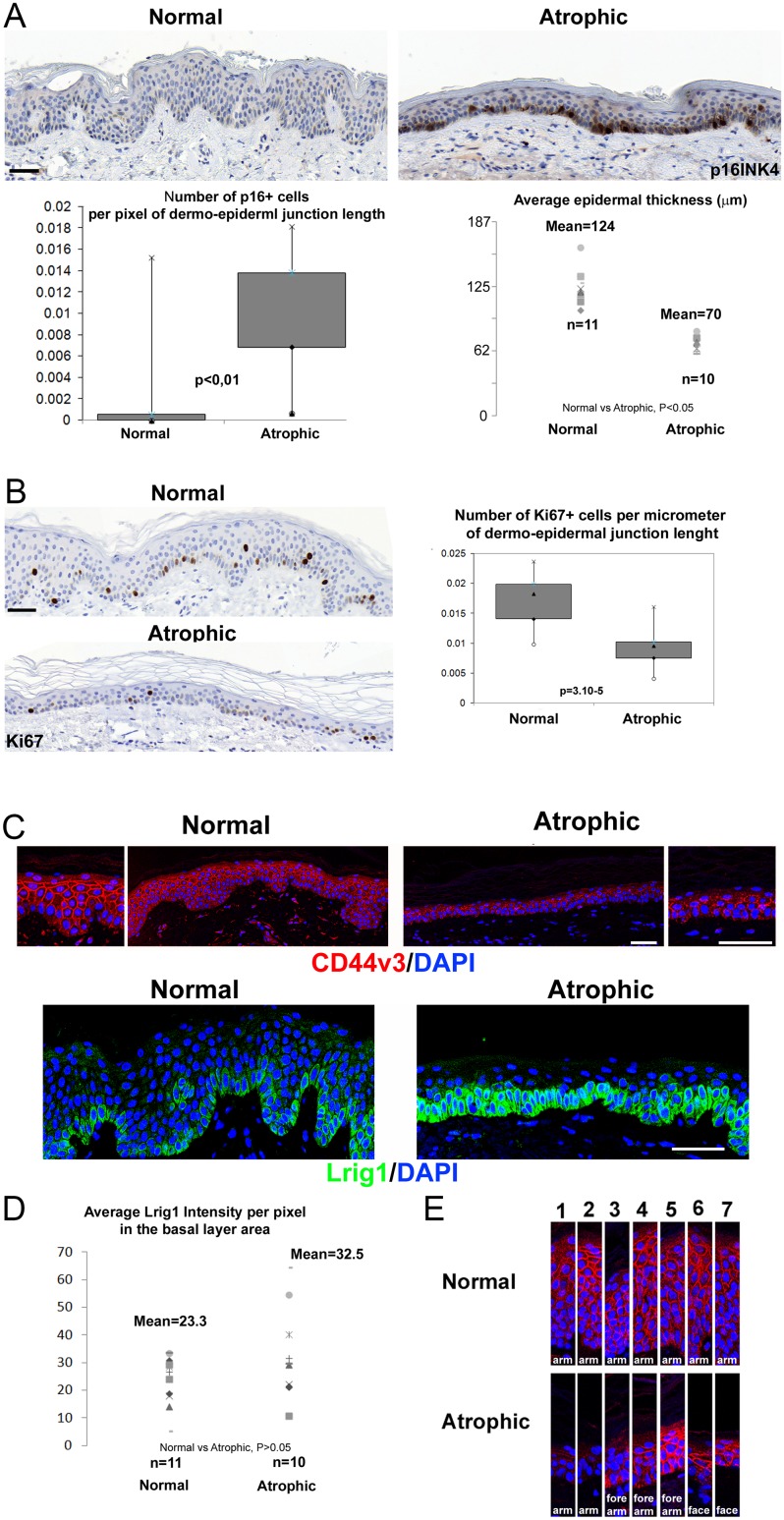
Loss of CD44v3 gradient and retention of Lrig1+ stem cells in senescent atrophic human epidermis. (A) p16INK4/CDKN2 staining in the epidermis of healthy (n = 11, average age of donors = 32yo with sd = 9) and senescent atrophic (n = 10, average age of donors = 76yo with sd = 13) donors, with quantification of p16INK4/CDKN2 for each group calculated as the number of p16 positive cells divided by the dermal-epidermal junction length and as the average of 3 microscope fields per donor, and the average epidermal thickness of 3 microscope fields per donor, calculated as the epidermal area divided by the length of the dermal-epidermal junction by field. (B) Representative CD44v3 (red) and Lrig1 (green) staining in the epidermis of the normal versus the atrophic group, DNA was counterstained with DAPI (blue). (C) Lrig1 intensity quantification for each donor calculated as the sum of pixel intensities in the green channel (256 levels) in the basal layer standardized to the total basal layer area measured in pixels. (D) CD44v3 staining samples from several individuals of each group, focusing on the CD44v3 expression gradient from the basal layer (down part) to upper differentiated layers (top part). Bar = 55μm.

**Fig 2:**
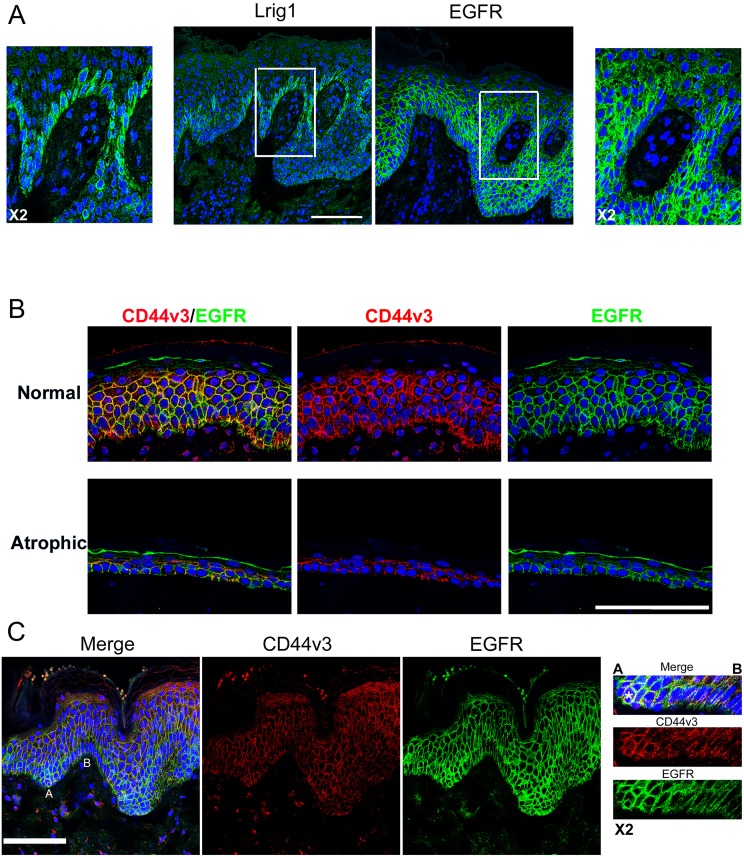
Lrig1 niche lacks EGFR and CD44v3 colocalizes with EGFR. (A) Lrig1 and EGFR staining (green) in two serial sections of normal human epidermis, blue = DNA stained with DAPI. (B) EGFR (green) and CD44v3 (red) co-staining in healthy and atrophic epidermis, (C) EGFR (green) and CD44v3 (red) co-staining in ridges of healthy epidermis, blue = DNA. Bar = 100μm.

A set of 11 biopsies of healthy human epidermis (HHE) were chosen in a skin biobank (anatomic locations: 10 = arm, and 1 = forearm). Another set of 10 biopsies with a significant atrophy of the epidermis were selected (anatomic locations: 4 = forearm, 2 = arm, 1 = neck, 3 = cheek). We confirmed the epidermal atrophy by measuring the epidermal thickness and confirmed the advanced stage of senescence with p16INK4/CDKN2 immunostaining (Fig 1A). This group was defined as the SAHE group. Proliferation was also downregulated, as shown by Ki67 stainings, in agreement with the SAHE phenotype (Fig 2B). In HHE, the expression pattern of CD44v3 showed to be stronger in the suprabasal layer. In SAHE, CD44v3 appeared downregulated almost in all arm/forearm locations (3 of 4 showed a less intense expression of CD44v3 in forearm, 2 of 2 showed poor expression of CD44v3 in arm location and cheek, and neck samples showed a more intense signal) (Fig 1C and 1E). CD44 expression was still preserved in chronically photoexposed skin, as previously shown [22], in contrast to the acute phase of UV exposure [23]. However, the increasing gradient of expression, which is always found in HHE, was lost in all SAHE samples. This reduced expression of CD44v3 has been previously reported [14,16]. In HHE, CD44v3 expression showed an increasing gradient of intensity from the basal layer to upper differentiated layers (Fig 1C and 1E). Surprisingly, Lrig1 was strongly expressed all along the basal layer of SAHE, contrasting with the usual pattern observed in HHE with clusters of keratinocytes highly positive for Lrig1 preferentially detected on the top of the rete ridges (Fig 1C and 1D). Thus, Lrig1+ cells are retained in SAHE and constitute the large majority of basal keratinocytes. As negative control for Lrig1 staining, we stained psoriatic epidermis (S1A Fig) and confirmed the total lack of Lrig1 signal, while expression of keratin 5 was normally detected in keratinocytes of the basal layer in SAHE, HHE and psoriasis (S1B Fig) [12].

Lrig1 was also co-stained with CD271, an epidermal stem cell marker reported to be downregulated in aged epidermis [24]. In our hands, in HHE, CD271 was co-localized with Lrig1 expression but appeared to mark a subset of cells within the Lrig1 compartment. Its expression was also detected in the hair follicle, where it was not always co-localized with Lrig1. In psoriasis, its expression was not detected in the IFE but was present in the hair follicle. Finally, in SAHE it was still detected but was less intense and rather expressed in the basal pole of keratinocytes, explaining probably why it may have been reported to be downregulated in aged epidermis (S2 Fig) [24].

**Fig 3:**
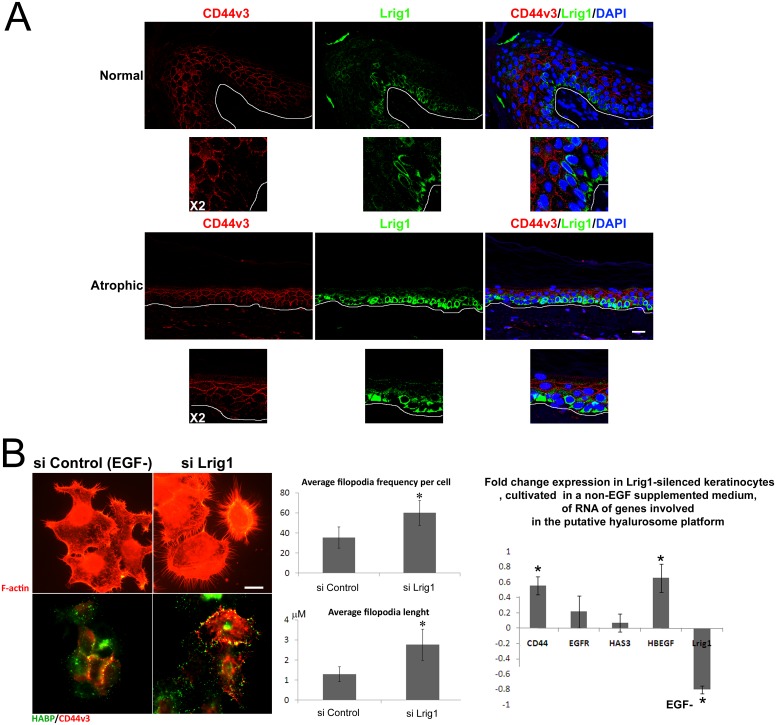
Lrig1 niche lacks CD44v3 expression. (A) Lrig1 (green) and CD44v3 (red) co-staining in healthy and atrophic epidermis, blue = DNA, (B) F-actin staining (red), and co-staining of HA (green) and CD44v3 (red) on keratinocytes transfected either with scramble siRNA or with Lrig1-targeting siRNA: quantification of filopodia length and frequency per cell calculated as the average value determined on 10 cells and fold change expression measured by quantitative PCR analysis of CD44, EGFR, HAS3, Hb-EGF and Lrig1 in similarly transfected keratinocytes (Y axis = fold change comparative expression between scramble siRNA-transfected keratinocytes and Lrig1-silenced keratinocytes. In both cases, expression was previously standardized according to the EEF1 housekeeping gene expression. Bar = 25μm. * indicates average values different from the scramble siRNA value with p<0.05.

Quantification of Lrig1 signal, measured as the amount of green intensity detected in the basal layer standardized to the basal layer area, indicated that the Lrig1 intensity was retained or tended to increase in SAHE basal cell layer, correlating with the loss of Lrig1 ^negative/low^ clusters. We then analyzed the differential expression of EGFR and CD44v3 in Lrig1+ and Lrig1- clusters in the basal layer of HHE (Fig 2). We confirmed the previously reported mutual exclusion of Lrig1 and EGFR expressions (Fig 2A) in serial sections of HHE [7]. CD44v3 was co-localized with EGFR (Fig 2B) and poorly expressed on the top of rete ridges where Lrig1^high^ clusters are located (Fig 2C). In epidermis of HHE and SAHE, CD44v3 expression was also excluded from cells expressing Lrig1 (Fig 3A). We further silenced the expression of Lrig1 in human keratinocytes in vitro by RNA interference (Fig 3B). In the Lrig1-silenced keratinocytes, filopodial structures were significantly induced together with the RNA expression of CD44 and HB-EGF. Silencing of the expression of the Lrig1 protein was also confirmed by western blot (S3 Fig).

### IFE of CD44KO mice is maintained by Lrig1-derived cells migrating from the hair follicle in homeostatic conditions

**Fig 4:**
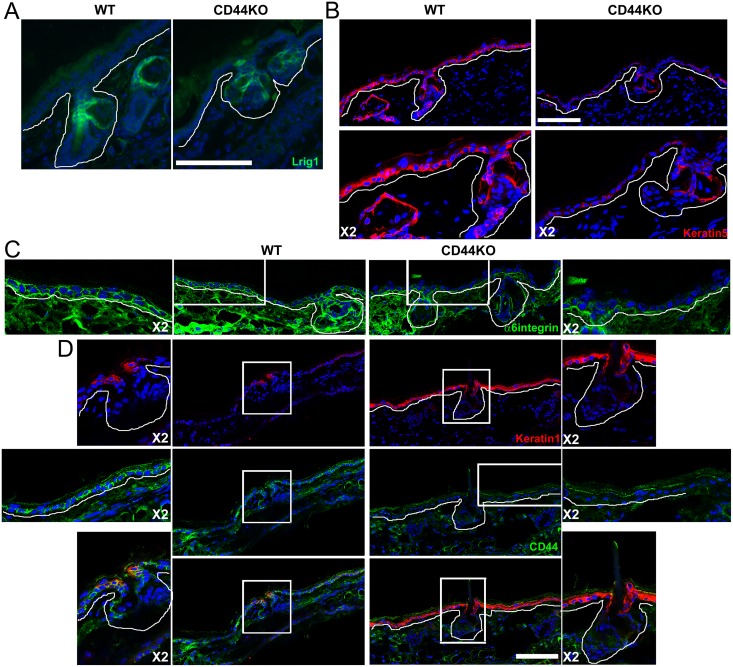
Loss of keratin 5 and α6–integrin expression in the basal layer of the IFE of CD44KO mice. (A) Lrig1 staining (green), (B) cytokeratin 5 staining (red), (C) α6-integrin staining (green), (D) cytokeratin 1 (red) and CD44 co-staining (green), in the ear epidermis of WT and CD44KO adult mice (>3 months). White line indicates the dermal-epidermal junction. Blue = DAPI. Bar = 87μm.

**Fig 5:**
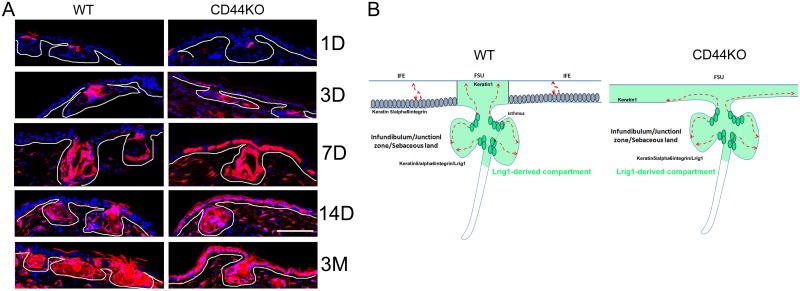
Lrig1+ cells feed the FSU only in WT and the FSU/IFE in CD44KO mice. (A) Tracing of the Lrig1-derived cells in the ear epidermis of WT versus CD44KO mice over a period of 3 months after injection of two doses of 2mg of Tamoxifen administered in one day. D = days and M = months after Tamoxifen injections. White line indicates the dermal-epidermal junction. (B) Schematical representation of WT versus CD44KO phenotype in the ear epidermis. Dark green cells = Lrig1+ cells, light green area = Lrig1-derived compartment. Bar = 87μm.

We further investigated the epidermal phenotype of CD44KO mice. We focused anatomically on ear for two reasons. First, this part of the body shows the most significant epidermal atrophy in adult animals (older than 3 months). Second, there are large areas of IFE, the homeostasis of which is known to rely on IFE basal keratinocytes that do not express Lrig1 and are therefore expected to divide according to an asymmetric model of division regulated by an autocrine Wnt signaling [4,5,25]. As expected, Lrig1 was expressed only in the junctional zone of the folliculo-sebaceous unit (FSU) in WT ears. This aspect was not altered in CD44KO mice (Fig 4A), even if the junctional zone seemed shorter than in WT mice. In homeostatic conditions, keratinocytes expressing Lrig1 in the junctional zone are expected to feed the sebaceous gland, junctional zone and infundibulum only. This defines two clusters: the FSU renewed by the Lrig1+ cells and the IFE that is renewed according to a stochastic model by the basal layer. We performed staining of cytokeratins 1&5 (K1&K5) and α6-integrin. As expected, K5 and α6-integrin were expressed in the entire basal layer of the IFE and the FSU in WT animals (Fig 4B and 4C). In contrast, in CD44KO mice, the expression of K5 and α6-integrin appeared strongly downregulated in the basal layer of the IFE, while their expression persisted in the FSU. In WT mice, K1 was not expressed in the ear IFE, and was only detected above the sebaceous gland, as previously reported [26] (Fig 4D). In contrast, K1 expression was detected in the infundibulum portion of the FSU as well as in the entire IFE basal layer in CD44 mice (Fig 4D). To confirm the hypothesis raised by these observations, that the two clusters identified in WT mice (FSU and IFE) are replaced by a unique cluster in CD44KO mice, we performed cell lineage tracing experiments. We confirmed that Lrig1+ cells only feed the sebaceous gland and the isthmus as well as the infundibular part of the FSU in WT mice. No Lrig1-derived keratinocyte (red cells) was detected in the IFE except in area very close to the FSU (Fig 5). In the opposite, in CD44KO mice, the sebaceous gland and isthmus were red, as well as the whole IFE, indicating that Lrig1+ keratinocytes also feed the IFE in CD44KO background.

### In SAHE CD44 expression and Wnt signaling are lost, while CD44 is required in vitro for Wnt signaling

**Fig 6:**
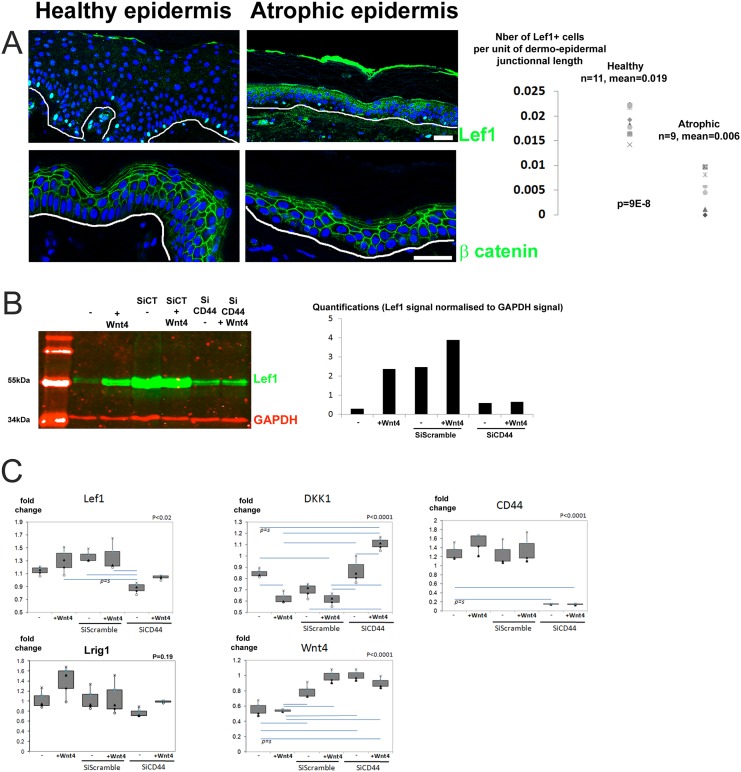
Lef1 expression is downregulated in senescent atrophic human epidermis and CD44 is required for Wnt signaling induction in human keratinocytes. (A) Lef1 and β-catenin staining in the epidermis of the healthy and senescent atrophic groups. Arrowheads indicate the Lef1+ cells with quantification of Lef1 for each patient of each group calculated as the number of Lef1+ cell standardized according to the dermal-epidermal junction length and as the average of 3 microscope fields per donor. White line indicates the dermal-epidermal junction. (B) Lef1(green) and GAPDH(red) western blot on human keratinocyte extracts, using Wnt4 (50ng/mL), scramble siRNA and siRNA CD44 with quantification of the Lef1 signal standardized to GAPDH. (C) Quantitative PCR analysis of the expression of Lef1, DKK1, CD44, Lrig1 and Wnt4 transcripts in keratinocytes treated as in (B). Blue horizontal bars indicate significant differences according the p values calculated with the ANOVA analysis of variance test with post hoc Tukey test, with P the overall significance value ANOVA value. 3 independent experiments were performed for each point. Bar = 60μm.

On behalf of the observations made in CD44KO mice, we hypothesized that a modification of the compartmentalization of the human basal layer may occur in human IFE in SAHE because of the severely disturbed expression of CD44. We focused on the Wnt signaling and the expression of Lef1, since Wnt autocrine signaling has been shown to regulate the IFE homeostasis in mouse skin [5]. Thus, we stained Lef1, as the expression of this transcription factor correlates with the activation of Wnt signaling, both in HHE and SAHE (Fig 6A). In SAHE, the frequency of keratinocytes positive for Lef1 in the basal layer was significantly reduced compared with normal epidermis. The expression of β-catenin was however not significantly affected, as shown by Lef1 and β-catenin costaining (S4 Fig). Co-staining of CD44 with Lef1 were also performed (S5 Fig).

We further looked whether CD44 downregulation might affect Wnt signaling, by silencing CD44 expression in human keratinocytes (Fig 6B and 6C). Normal keratinocytes appeared to be able to respond to Wnt4, as Lef1 expression was increased when incubated with human recombinant Wnt-4 peptide (50ng/mL) for 24h, showing an induction of the Wnt pathway (Fig 6B). Unfortunately transfection with a scramble siRNA was sufficient to induce a strong Lef1 expression, making difficult to induce Lef1 expression with exogenous Wnt4 after transfection. Quantitative PCR experiments further confirmed that transfection with scramble siRNA induced transcription of Lef1 and Wnt4 in agreement with western blot analysis (Fig 6C). In keratinocytes transfected with CD44 siRNA, Lef1 remained however poorly expressed as shown by western blot analysis and by Lef1 RNA expression. QPCR also showed that upon CD44 silencing, DKK1 transcript significantly increased while Wnt4 expression was still induced by the transfection. Expression of Axin2, EGFR, c-myc, β-catenin, Lrig1 and LRP6 transcripts were also analyzed but showed no significant variation. All together, this indicates that in the absence of CD44, Wnt signaling is hardly inducible. Inhibition of Wnt signaling by DKK1, a known inhibitor of the Wnt pathway, might explain this observation.

## Discussion

### From two self-renewing clusters in WT mice (IFE and FSU) to a unique FSU compartment in the epidermis of CD44KO mice

In normal mouse epidermis, at least three independent compartments have been identified in homeostatic conditions: (i) the IFE has been shown to self-renew according a stochastic model of division and to be regulated by an autocrine Wnt signaling taking place in the basal layer [4,5,27], (ii) the variable part of the hair follicle is renewed by Lgr5-expressing keratinocytes located in the bulge, (iii) and the junctional zone, infundibulum and the sebaceous gland are renewed by the Lrig1-expressing cluster of keratinocytes, located in the isthmus [2].

In adult CD44KO mice, keratin 5 and α6-integrin expression disappeared in the IFE, while keratin 1 expression was found in the basal keratinocytes of the IFE. This suggests a switch in the basal layer of the IFE secondary to CD44 loss. Cell lineage tracing experiments in the CD44KO mice confirmed this switch, as IFE was renewed by Lrig1-derived keratinocytes that migrate from the isthmus, where the Lrig1+ cells are located, to the basal layer of the IFE (Fig 4). This demonstrates that in CD44KO mice, IFE rapidly lost its self-renewing capacity, a loss which is however rescued by Lrig1+ stem cells located in the isthmus of the hair follicle. The CD44KO mice were first reported to present no obvious phenotype and to reproduce normally [18]. At the cutaneous level, the expression of differentiation markers were reported to be disturbed and the assembly of tight-junctions was shown to be impaired [20,28]. A delayed wound healing and hair regrowth was also reported, pointing out a disturbed epidermal homeostasis [13,14,15,21]. It appears that the homeostasis of IFE is abrogated or at least severely impaired in ears of the CD44KO mice. Then our data shows that CD44 would be required for IFE stem cells proliferation, but dispensable for the Lrig1+ stem cells that renew the constant part of the HF and the SG. Importantly, IFE renewal was previously shown to be regulated by the Wnt signaling pathway [3,5], suggesting that CD44 may be involved in this regulatory pathway.

### Similarities between SAHE and the aging CD44KO mouse epidermis

The presence of Lrig1^high^/EGFR^low^ and Lrig1^low^/EGFR^high^ clusters in basal keratinocytes of human epidermis is well established [7,29]. Our results add a third marker, CD44v3 which is expressed in the Lrig1^low^/EGFR^high^ clusters, thus defining two basal layer clusters: the Lrig1^high^/CD44v3^low^EGFR^low^ and the Lrig1^low^/CD44v3^high^EGFR^high^.

These two clusters are consistent with in vitro observation on keratinocytes, as CD44 and EGFR are both co-expressed on the top of filopodial protrusions [13]. In addition, Lrig1 silencing tended to induce a strong increase of filopodia as well as an increase in CD44 and HB-EGF expression, which are both involved in filopodia structures and the putative hyalurosome platform [13]. The fact that Lrig1 may be an inhibitor of filopodia structures where EGFR signaling is induced makes sense considering the EGFR-inhibition activity of Lrig1 [30].

Besides the Lrig1/EGFR axis, Wnt signaling also probably contributes to human epidermal homeostasis. Thus, lef1 expression makes clear that Wnt signaling is highly active in human epidermal basal layer. CD44 seems to be involved in Wnt signaling, since (i) the ear IFE of CD44KO mice lost its ability to self-renew, a property linked to Wnt autocrine signaling (Figs 4 and 5) and (ii) since CD44 appeared to be necessary for Wnt activation in human keratinocytes in vitro via dkk1 (Fig 6). Thus, Wnt signaling may rather take place in the Lrig1^low^/CD44v3^high^EGFR^high^ cluster in the basal layer of human epidermis. By analogy with the mouse model, this cluster may constitute a self-renewing compartment active in homeostasis, while the Lrig1^high^/CD44v3^low^EGFR^low^ cluster may operate as a reservoir compartment of quiescent cells active upon injury. Our data indicate that in absence of CD44, expression of DKK1, a strong inhibitor of the Wnt pathway, is induced and it may be the reason why CD44-silenced keratinocytes do not respond in vitro to exogenous Wnt4 peptide. DKK1 acts by isolating LRP6 co-receptor from the receptor Frizzled. Without LRP6, frizzled receptor becomes unable to bind Wnt ligands and to initiate Wnt-signaling. CD44 may then operate as a co-receptor contributing to the binding of the family of Wnt ligands to the LRP6/Frizzled receptor complex. A similar mechanisms has already been reported in the epithelium intestine, where CD44 showed to positively modulate LRP6 membrane localization and availability for its association with Frizzled [31].

### Downregulation of Wnt signaling related to atrophy in senescent human epidermis

We hypothesized that the downregulation of Wnt signaling may be involved in the SAHE phenotype. This was supported by (i) the highly disturbed CD44 expression in SAHE, CD44 appeared to be necessary for Wnt signaling in vivo in mouse and in vitro in human keratinocytes, and (ii) the finding that Lef1 is significantly downregulated in SAHE (Figs 1 and 6). Thus, the IFE atrophy observed in CD44KO mice may be a murine model of human SAHE. This is consistent with a recent report where CD44 is a positive modulator of the Wnt pathway in intestinal cells [32]. CD44 may be a regulator of Wnt signaling in the basal layer of human epidermis. Together with its ability to modulate EGFR [13], the Wnt signaling could be another molecular pathway by which CD44 regulates epidermal growth.

We further speculate, that in SAHE, Lrig1^high^/EGFR^low^CD44v3^low^ clusters may extend while conversely the Lrig1^low^/CD44v3^high^EGFR^high^ clusters may regress. Retention of Lrig1^high^ keratinocytes in senescent human epidermal basal layer, as shown in Fig 1, may be the phenotype of an epidermal homeostasis rescued by Lrig1-derived cells in the absence of Wnt signaling. A hallmark of epidermal aging may then be a downregulation of the Wnt pathway induced by a downregulation of CD44 expression. The latter is also a HA receptor, pointing CD44 as a remarkable platform connecting the extra-cellular-matrix (ECM) with the Wnt pathway. ECM might then affect cell renewal in epidermis via CD44, which could be one of the mechanisms involved in epidermal aging. However, Lrig1+ stem cells may avoid a dramatic failure of epidermal homeostasis, as these cells showed to be preserved in human senescent epidermis and were also shown to not be affected in CD44KO mice.

## Supporting Information

S1 Text Quantitative PCR protocols and probes. (TIF)

S1 Fig Staining of Lrig1 and keratin 5 in normal, atrophic and psoriatic human epidermis. Comparative expression of Lrig1 (green) (A) and keratin 5 (red) (B) in normal, atrophic and psoriatic human epidermis, Blue = DAPI. Bar = 100μm.
(TIF)

S2 Fig Lrig1 and CD271 co-staining in normal epidermis, hair follicles, atrophic and psoriatic human epidermis. (A, B, C, F) Co-staining of CD271 (green) and Lrig1 (red) in the interfollicular epidermis and the hair follicle of normal or psoriatic scalp skin, (D, E) single CD271 staining (green) in the interfollicular epidermis and the hair follicle of normal skin, (G) co-staining of CD271(green) and Lrig1 (red) in normal and senescent atrophic epidermis of the arm. Blue = DAPI. Bar = 75μm.
(TIF)

S3 Fig Lrig1 Western-Blotting in Lrig1-silenced keratinocytes. Lrig1 western blotting of protein extracts from human primary keratinocytes transfected with control SiRNA and Lrig1 SiRNA, with quantifications made on 3 distinct experiments.
(TIF)

S4 Fig β-catenin co-staining with Lef1 in healthy and atrophic epidermis. Bar = 100μm.
(TIF)

S5 Fig Colocalization of CD44v3 and Lef1 in human epidermis. (TIF)

## Abbreviations

FSU: folliculo-sebaceous unit
HA: Hyaluronate
HHE: healthy human epidermis
IFE: interfollicular epidermis
SAHE: senescent atrophic human epidermis
