# Supplementary figures and images for: Senescent Atrophic Epidermis Retains Lrig1+ Stem Cells and Loses Wnt Signaling, a Phenotype Shared with CD44KO Mice
Source: PLoS One. 2017 Jan 18;12(1):e0169452. doi: 10.1371/journal.pone.0169452 (PMC5242443; doi:10.1371/journal.pone.0169452)

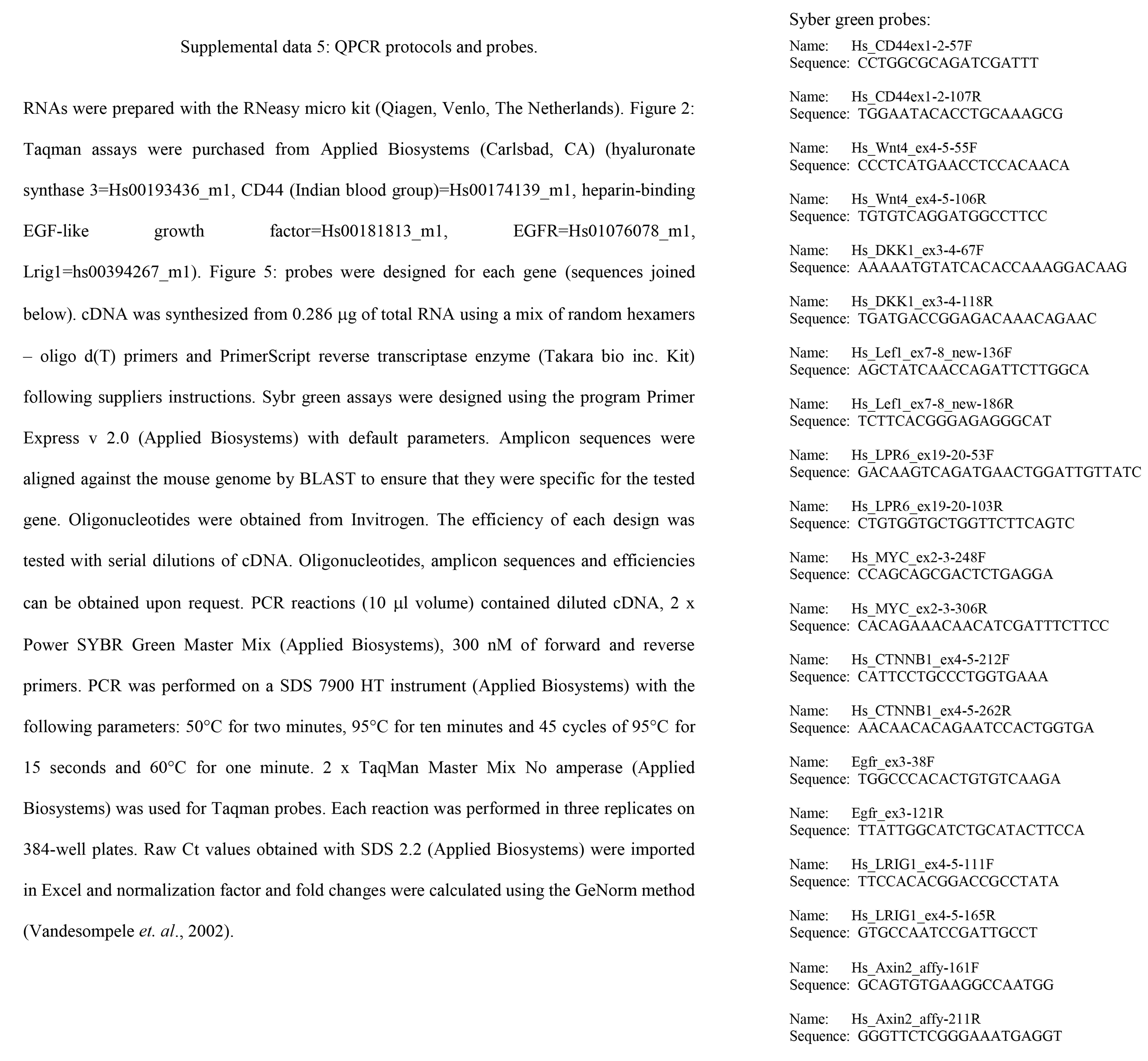

Supplement: S1 Text — (TIF) [file pone.0169452.s001.tif]

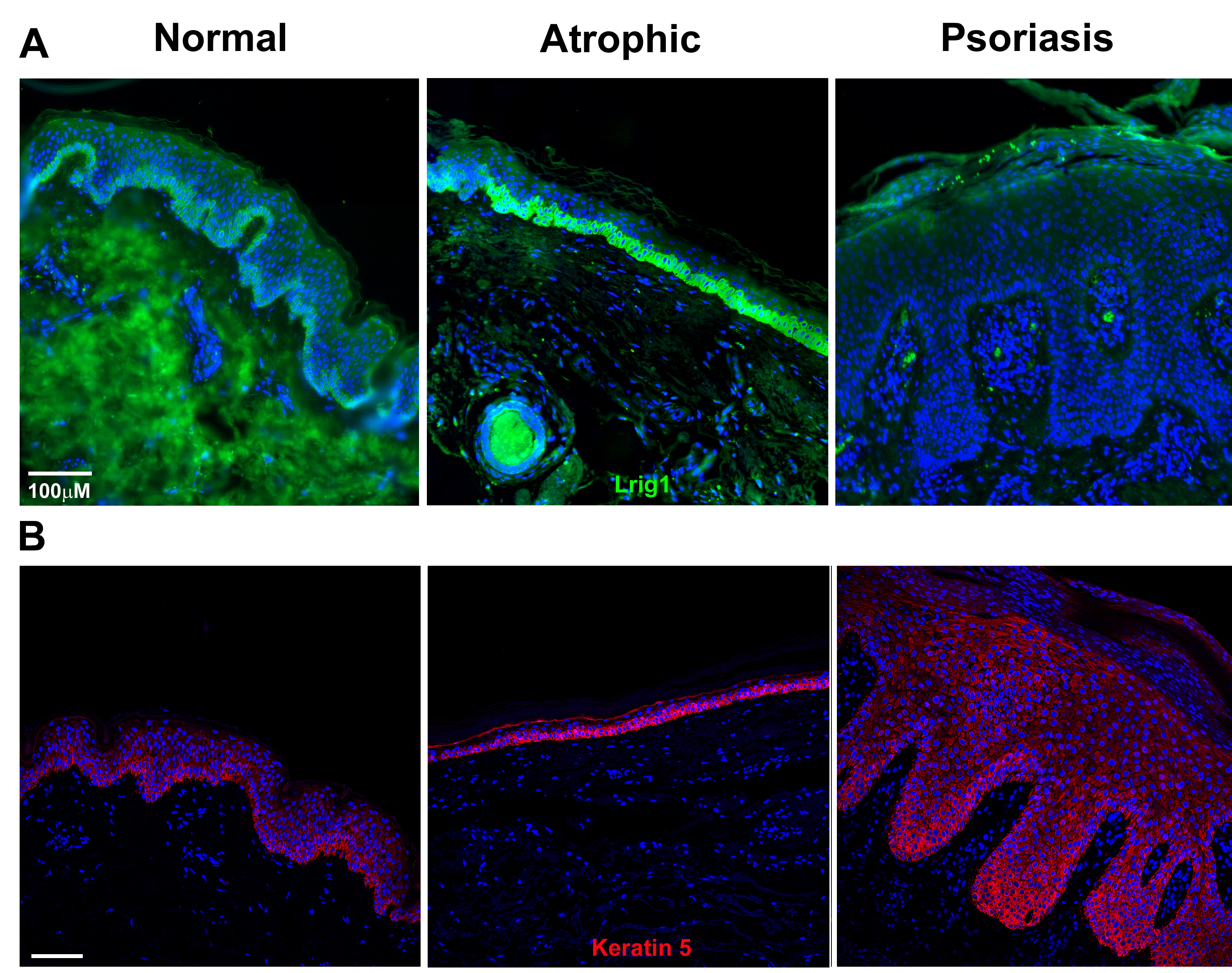

Supplement: S1 Fig — Comparative expression of Lrig1 (green) (A) and keratin 5 (red) (B) in normal, atrophic and psoriatic human epidermis, Blue = DAPI. Bar = 100μm. (TIF) [file pone.0169452.s002.tif]

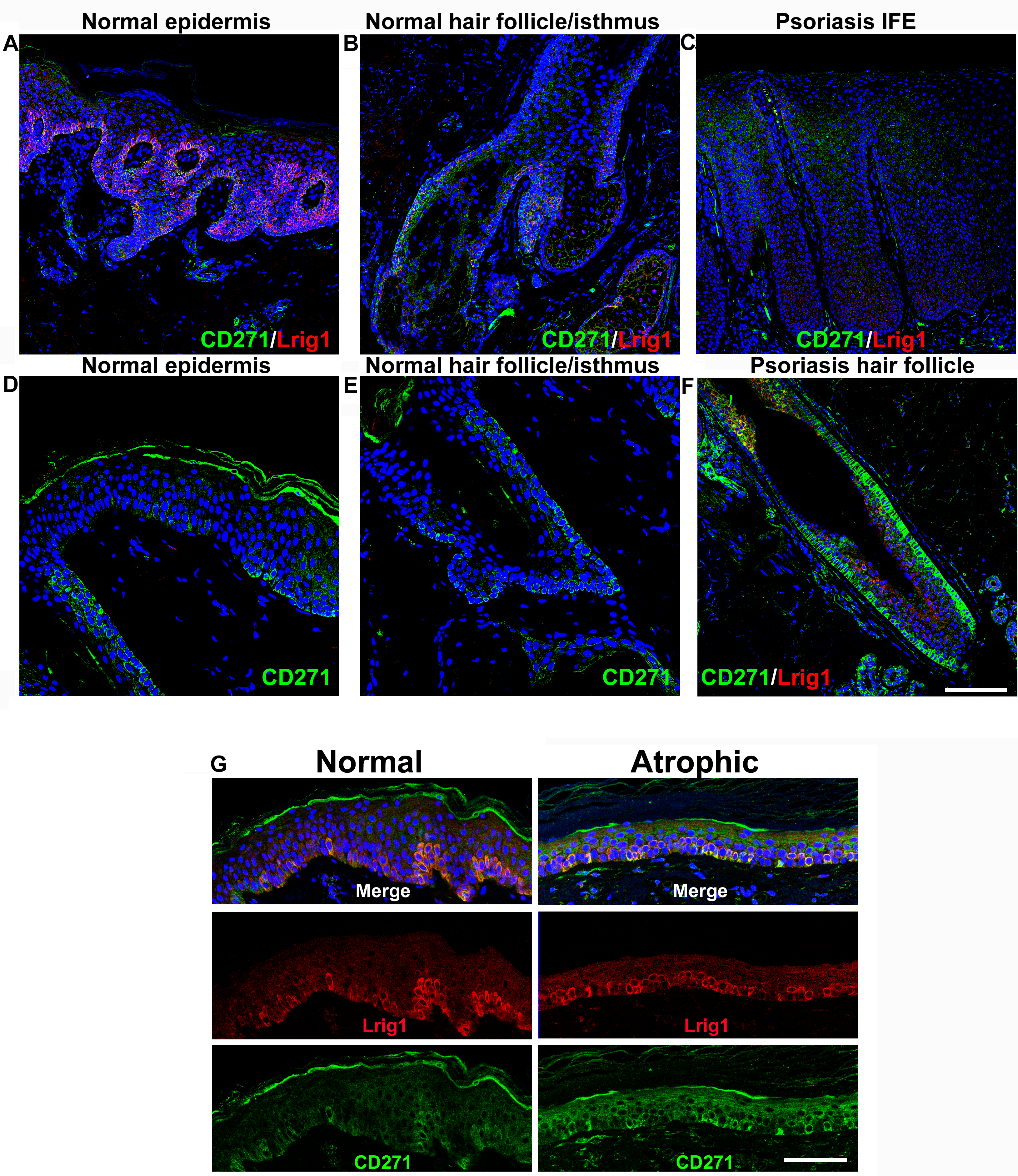

Supplement: S2 Fig — (A, B, C, F) Co-staining of CD271 (green) and Lrig1 (red) in the interfollicular epidermis and the hair follicle of normal or psoriatic scalp skin, (D, E) single CD271 staining (green) in the interfollicular epidermis and the hair follicle of normal skin, (G) co-staining of CD271(green) and Lrig1 (red) in normal and senescent atrophic epidermis of the arm. Blue = DAPI. Bar = 75μm. (TIF) [file pone.0169452.s003.tif]

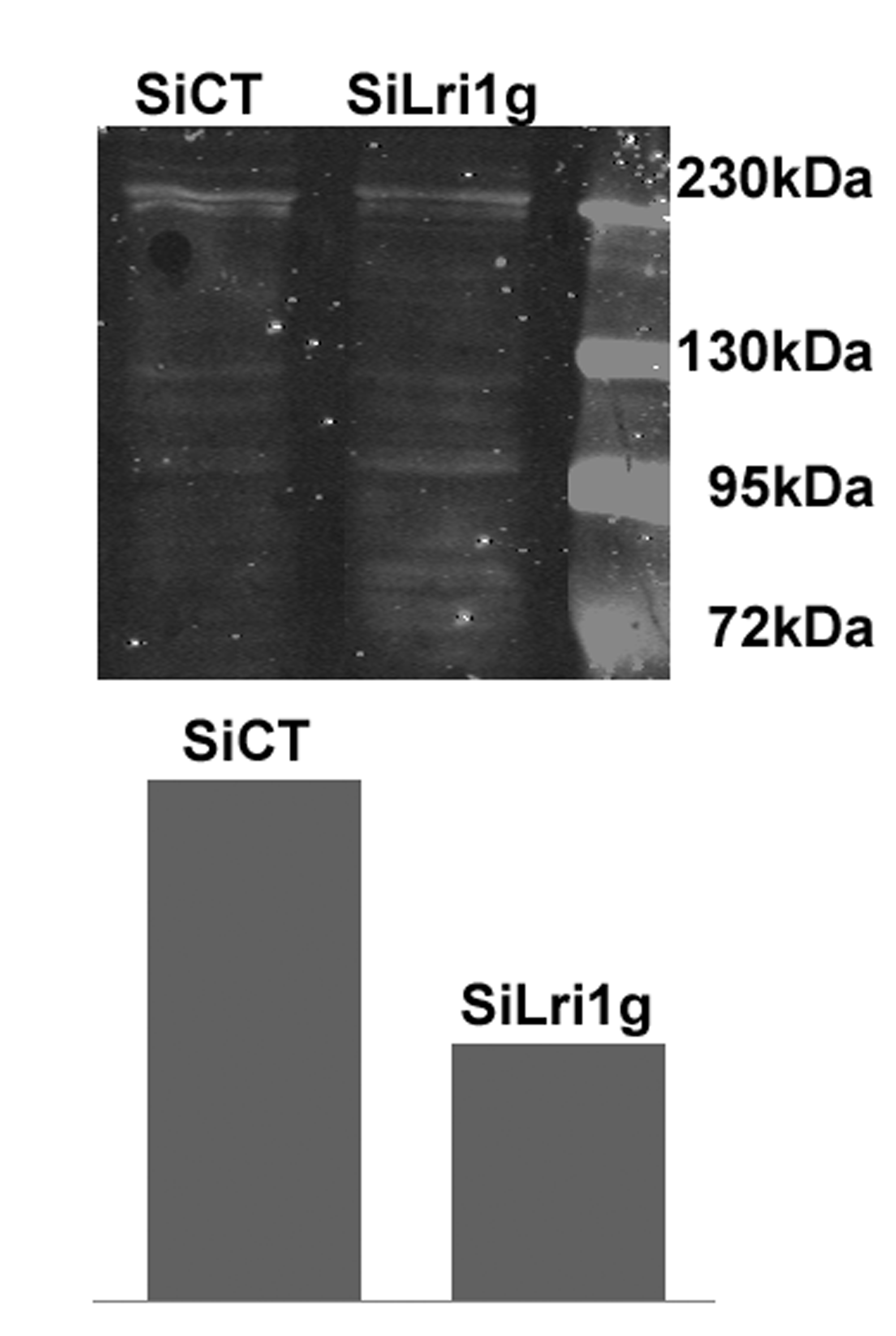

Supplement: S3 Fig — Lrig1 western blotting of protein extracts from human primary keratinocytes transfected with control SiRNA and Lrig1 SiRNA, with quantifications made on 3 distinct experiments. (TIF) [file pone.0169452.s004.tif]

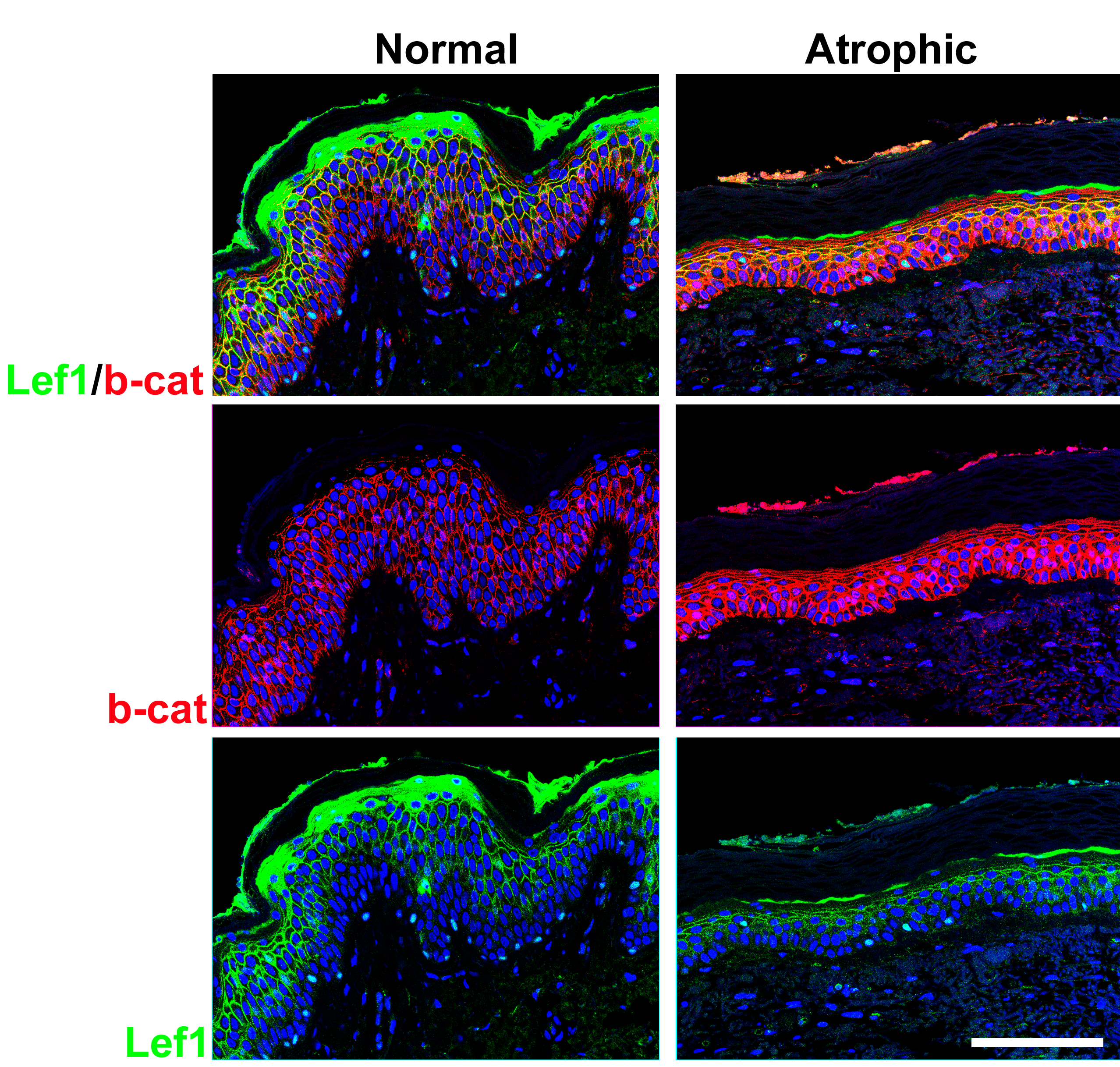

Supplement: S4 Fig — Bar = 100μm. (TIF) [file pone.0169452.s005.tif]

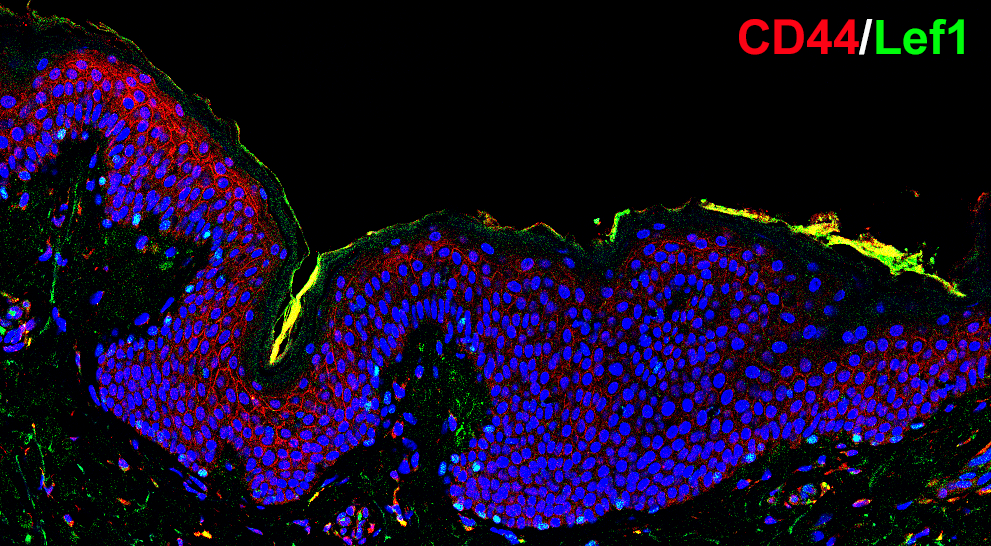

Supplement: S5 Fig — (TIF) [file pone.0169452.s006.tif]
